# Abstracts of the 3^rd^ International Conference on Cancer Cachexia, September 23–25, 2016 in Washington, DC, USA

**DOI:** 10.1002/jcsm.12186

**Published:** 2017-02-27

The 3rd International Conference on Cancer Cachexia titled, “*Advancing Molecular Mechanisms and Therapies*” took place on September 23–25, 2016, in Washington, DC. This meeting followed the 1st conference in 2012 in Boston, USA, and the 2nd in 2014 in Montreal, Canada. The goal for the 3rd meeting was to continue to advance our understanding of the basis of cancer‐induced cachexia by discussing new topics and unpublished data. In addition, we placed greater emphasis in this meeting on therapies and the education of clinical trial design and outcome measures. The meeting included new sessions to discuss cross‐talk pathways between tissues, new animal models of cancer cachexia, and interpretations of large data sets. In addition, the meeting incorporated a half‐day session on the design of cancer cachexia clinical trials, an event that was well attended and led to engaging conversations among attendees. Highlights from the conference included our Keynote Speaker, Dr Vickie Baracos from the University of Alberta, a presentation from a patient advocate, Mr David Kochanczyk, and a special lecture from Dr Douglas Lowy, Director of the National Cancer Institute, USA. We also presented our personal reflections to remember our close colleague, Dr Ken Fearon who truly was a maverick in the field.

Below represent some of the abstracts presented at the conference supporting both oral and poster presentations. We sincerely thank the editors of the Journal of Cachexia, Sarcopenia and Muscle (JCSM) for allowing the submission and publications of these abstracts.

2016 Cancer Cachexia Conference Planning Committee

**Denis C. Guttridge, PhD**

The Ohio State University, Columbus, Ohio, USA

**Josep Argiles, PhD**

University of Barcelona, Spain

**Teresa A. Zimmers, PhD**

Indiana University School of Medicine, Indiana, USA

## The concurrent advancement of cancer cachexia science and oncology clinical practice

Vickie E Baracos

*University of Alberta, Edmonton, AB, Canada*

Cancer treatment planning is based on assessments of the degree to which the disease has spread from its primary site, of the patient's fitness to tolerate treatments, which are increasingly aggressive, and estimation of expected survival. Information about cachexia can add materially to making these assessments, because defined cachexia criteria are independently prognostic of survival as well as complications of cancer treatments. The prognostic cachexia diagnostic criteria are weight loss, inflammation, reduced food intake and computed‐tomography (CT) – defined muscle cross‐sectional area, muscle radiodensity and muscle and fat loss. Powerful discrimination of survival, complications of cancer surgery and toxicity of systemic cancer therapy is provided by the CT‐defined metrics. The availability and applicability of diagnostic imaging in clinical oncology has added to a rapid recent adoption of CT‐defined cachexia in clinical oncology research. Catabolic losses/depletion of muscle and fat is precisely quantified, and these show strong independent relationships with outcomes. A compelling new theme in this area is the identification muscle with reduced radiodensity (indicative of steatosis) as a predictor of survival and cancer surgery complications. Quantification of a patient's cachexia characteristics can have immediate clinical utility in the estimation of survival, disease spread and risk of complications. Furthermore, body composition features may provide a means of more rigorously adjusting chemotherapy treatments to characteristics of individual patients. Relevant clinical tools to acquire and deploy cachexia‐related information must be put into the hands of oncologists and cancer radiologists.

In the longer term, research into cachexia mechanisms and therapeutics holds the potential to provide clinical and even survival benefit to patients with cancer. Scientists are increasingly viewing cachexia as a distinct, potentially treatable condition. For example, pharmacological blockade of the myostatin/activin‐ActRIIB pathway prevented and reversed the loss of muscle mass and strength in cancer cachexia and also to markedly prolong the lifespan of animals with cancer‐associated muscle loss. Significantly increased survival has now been demonstrated multiple times in animal models with experimental cachexia therapeutics. Clinical translation of such findings lies in the future. However, we now have several lines of evidence that cancer patients have anabolic potential. Activation of muscle protein synthesis in response to the provision of specific mixtures of amino acids and other nutrients has been shown. Drugs induce significant gains of muscle mass in patients even with advanced cancer including *Anamorelin*® (Phase III), Enobosarm® (Phase II) and Selumetinib® (Phase II). This evidence contradicts the widely held misconception that cachexia is irreversible and holds the promise that we may achieve important clinical benefits through targeted cachexia therapy.

## Loss of adipose tissue is a predictor of survival outcomes in lymphoma treated with hematopoietic stem cell transplantation from an allogeneic donor

Asmita Mishra ^1^, Kerry Thomas^1^, Vickie Baracos^2^, Kevin Bigam^2^ and Martine Extermann^1^

^1^ *H. Lee Moffitt Cancer Center, Tampa, Florida, USA;* ^2^ *University of Alberta Canada, Edmonton, Alberta, Canada*

Recent findings suggest that computed‐tomography (CT)–defined features of body composition (ie skeletal muscle, fat amount and distribution) predict for cancer‐associated survival. While many studies have evaluated skeletal muscle, the significance of adipose tissue is otherwise unknown for hematologic malignancies.

We performed a single institution, retrospective review of lymphoma patients who received allogeneic hematopoietic stem cell transplantation (allo‐HCT) between 2005 and 2016. Lymphoma patients were chosen for this analysis as CT imaging is a part of routine disease status evaluation. Patient demographics, including sex, Karnofsky performance status (KPS), transplantation‐specific comorbidity index (Sorror et al), chemotherapy intensity, and body mass index (BMI) were reviewed. Pre‐transplant CT scans done 30 days +/− 30 days as per protocol. Post‐transplant CT scans done 90 days +/−30 after transplantation per physician preference. Scans were analyzed according to established conventions, using a single CT slice at the 3rd lumbar for cross sectional area of skeletal muscle and adiposity tissue.

Of the 96 patients reviewed, 56% were male (*n* = 54), with median age of 53.6 years (range 23.5–68.1) and mean BMI of 30.3 +/− 4.2 kg/m^2^. Females in this cohort (*n* = 42, 44%) had a median age of 53.2 (range 27.7–68.6) with mean BMI of 27.5 +/− 4.1 kg/m^2^. Majority of the patients received myeloablative chemotherapy regimen (67%) with KPS ≥ 90 (79%). Overall survival (OS) was evaluated in relation to CT defined characteristics. When comparing before and after allo‐HCT, patients lost on average skeletal muscle (−11.6 cm^2^, range: −50.3–29.6) and total adiposity (−23.0 cm^2^, range: −225.2 to 134.0). Fat loss of more than 25% between CT scans predicted for worse survival. The median survival for the high adipose loss patients (*n* = 12) was 9.3 months [95% CI 4.84 – not reached] as compared to those that loss less fat (*n* = 84) with a median survival was not reached [77.6 – not reached) (*p* = 0.0011). In addition to having high fat loss, the poor prognosis group also had loss of muscle with high adipose loss group having 11% of cross‐sectional skeletal muscle as compared to 8% in low adipose loss group.

Loss of adiposity after transplantation is an inexpensive and powerful tool that can complement other prognostic clinical outcomes after transplantation.

## Muscle function matters: Role of the tumor‐bone microenvironment in regulating muscle function in cancer

David Waning, Khalid S Mohammad, Steven Reiken, Wenjun Xie, Daniel Andersson, Antonella Chiechi, Laura A Wright, Jenna N Regan, Trupti Trivedi, Xu Cao, G David Roodman, Andrew R Marks and Theresa A Guise

*Department of Medicine at Indiana University (IUPUI) and the Department of Physiology at Columbia University, USA*

Cancer‐associated weakness is a therapeutic challenge. We found skeletal muscle weakness in six mouse models of human osteolytic bone metastases [breast (3), lung (2), prostate (1)], and in multiple myeloma, but not in mice without cancer in bone, implicating the tumor‐bone microenvironment in muscle weakness. Tumor‐induced bone destruction released TGF‐β. TGF‐β upregulated NADPH oxidase 4 (Nox4) that oxidized skeletal muscle proteins, including the ryanodine receptor/calcium release channel (RyR1). Humans with breast or lung cancer bone metastases also had oxidized skeletal muscle RyR1. Oxidized RyR1 leaked calcium causing muscle weakness. Inhibiting RyR1 leak, TGF‐β signaling, TGF‐β release from bone (with bisphosphonate zoledronic acid) or Nox4 all improved muscle function. Increasing muscle mass alone, with activing receptor antibody, did not improve muscle function. Skeletal muscle weakness, increased Nox4 and oxidation of RyR1 were present in a mouse model of Camurati–Engelmann disease, a non‐malignant metabolic bone disorder associated with increased TGF‐β and high bone turnover. Thus, bone‐derived TGF‐β contributes to muscle weakness by decreasing calcium‐induced muscle force production, and this may occur even before the loss of muscle mass. These findings indicate that important cross‐talk exists between bone (via bone destruction) and muscle that could be targeted to prevent both bone loss and muscle weakness.

## The activation of the SDF1/CXCR4 pathway in muscle retards atrophy during cancer cachexia

Giulia Benedetta Martinelli^1^, Davide Olivari^1^, Laura Talamini^1^, Stewart H. Lecker^2^, Linda Ottoboni^3^, Andrea Resovi^4^, Raffaella Giavazzi^1^, Luigi Cervo^5^ and Rosanna Piccirillo ^1^

^1^ *Department of Oncology and Neuroscience, IRCCS ‐ Mario Negri Institute for Pharmacological Research, Milan, Italy;* ^2^ *Beth Israel Deaconess Center, Boston, MA, USA;* ^3^ *San Raffaele Scientific Institute, Milan, Italy;* ^4^ *Department of Oncology, Tumor Angiogenesis Unit, IRCCS ‐ Mario Negri Institute for Pharmacological Research, Bergamo, Italy;* ^5^ *Neuroscience, IRCCS ‐ Mario Negri Institute for Pharmacological Research, Milan, Italy*

Cancer cachexia is a life‐threatening syndrome that affects most patients with advanced cancers and involves severe body weight loss, with rapid depletion of skeletal muscle. No effective treatment is available.

We analyzed microarray datasets to identify a subset of genes whose expression is specifically altered in cachectic muscles of Yoshida hepatoma‐bearing rodents, but not in those with diabetes, disuse, uremia or fasting. Ingenuity Pathways Analysis indicated that three genes belonging to the C‐X‐C motif chemokine receptor 4 (CXCR4) pathway were downregulated only in muscles atrophying because of cancer: stromal cell‐derived factor 1 (*SDF1*), adenylate cyclase 7 (*ADCY7*), and p21 protein‐activated kinase 1 (*PAK1*). Notably, we found that, in the Rectus Abdominis muscle of cancer patients, the expression of *SDF1* and *CXCR4* was inversely correlated with that of two ubiquitin ligases induced in muscle wasting, *atrogin‐1* and *MuRF1*, suggesting a possible clinical relevance of this pathway. The expression of all main *SDF1* isoforms (α, β, γ) also declined in Tibialis Anterior muscle from cachectic mice bearing murine colon adenocarcinoma or human renal cancer and drugs with anticachexia properties restored their expression. Overexpressing genes of this pathway (in other words, *SDF1* or *CXCR4*) in cachectic muscles increased the fiber area by 20%, protecting them from wasting. Similarly, atrophying myotubes treated with either SDF1α or SDF1β had greater total protein content, resulting from reduced degradation of overall long‐lived proteins. However, inhibiting CXCR4 signaling with the antagonist AMD3100 did not affect protein homeostasis in atrophying myotubes, whereas normal myotubes treated with AMD3100 showed time‐ and dose‐dependent reductions in diameter, until a plateau, and lower total protein content. This further confirms the involvement of a saturable pathway (i.e. CXCR4). Overall, these findings support the idea that activating the CXCR4 pathway in muscle suppresses the deleterious wasting associated with cancer.

## Canine model – Safety and efficacy of TCMCB07, a novel melanocortin‐4 receptor antagonist

Sandra M Bechtel^3^, Daniel L Marks^1^, Xinxia Zhu^1^, Kimberly A Selting^3^, Brian K Flesner^3^, Debbie J Tate^3^, Brittany M Angle^2,3^, Carolyn J Henry^3^, Jeffrey N Bryan^3^, Kenneth A Gruber^2,3^, DG Scholten^2,3^ and Michael F Callahan ^2,3^

^1^ *Oregon Health and Science Univ;* ^2^ *Tensive Controls and;* ^3^ *Univ of Missouri‐Columbia, USA*

CNS Melanocortin (MC) pathways play a key role in nutrient intake, energy expenditure and wasting associated with numerous disease states. MC antagonists attenuate cancer, renal, cardiac and LPS‐induced cachexia. TCMCB07 shows efficacy and safety in rodent renal and cancer‐induced cachexia, maintaining food intake, body weight and lean body mass. The current study examined safety, pharmacokinetics and efficacy of TCMCB07 in normal dogs and companion dogs with cachexia.

In a one‐arm open‐label study, TCMCB07 was given subcutaneous (s.c.) at 2.25 and 0.75 mg/kg/d for 28 d to 5 healthy beagles. Body weight, CBC, systolic arterial pressure and 24 hour Holter ECG were obtained at baseline, days 1, 5 and 28. Blood was sampled at baseline, 30 m, 1, 2, 4, 12 and 24 hours for assay of TCMCB07. In the companion trial, dogs with cachexia (>5% weight loss) were treated at 0.75 mg/kg/d for 28 days and offered extended (compassionate) use at the end of the trial. Tufts Animal Care and Condition scale and body weight were obtained weekly. A CVC, blood chemistry and urinalysis were performed on day 0, 14 and 28 with blood pressure and ECG Holter performed on days 0 and 28.

Plasma TCMCB07 peaked at 1–2 hours after s.c. administration and were near the LOD by 24 hours. During 28 day dosing, dogs gained weight at both doses (9 and 13% increase at low and high dose, respectively). A modest eosinophilia was observed at 28 days. Two companion dogs enrolled in the trial have shown increases in body weight (11–12%) and improvements in body condition score either during the formal trial or under compassionate use. One dog has now completed 4 months of compassionate use. There were no apparent changes in eosinophils, and no significant arrhythmias noted.

TCMCB07 appears to be safe in normal dogs, and early results in companion dogs thus far support both safety and efficacy.

## Effects of postoperative complications on the weight of patients suffering from pancreatic cancer

Marc Martignoni^1^, Tara Müller^2^ and Andreas Prischenk ^3^

^1^ *Department of Surgery, University Hospital Klinikum Rechts der Isar, Munich, Germany;* ^2^ *Department of Surgery, University Hospital Klinikum Rechts der Isar, Munich, Germany;* ^3^ *Technical University of Munich, Germany*

**Introduction:** Pancreatic cancer is the cause of every fifth cancer‐related death in the western world. The prognosis is extremely poor with a five‐year‐survival rate of three to five percent. About 80 percent of all pancreatic ductal adenocarcinoma patients suffer from cachexia, which lowers overall survival significantly. Consequently, weight loss undeniably affects the prognosis. However, the question arises whether the patients' weight is affected by postoperative complications.

**Material and methods:** In our study, we analyzed the data of 44 patients with pancreatic cancer, who underwent surgery at our clinic between 5/2008 and 7/2013. Postoperative complications in the first 30 days were rated after the Clavien–Dindo classification. Postoperative complications, which occurred after 30 days but still within the first year after surgery, such as a follow‐up surgery, were defined as late complications. The patients' weight was monitored after six and twelve months.

**Results:** Patients who suffered from postoperative complications had a tendency towards weight loss after twelve months. However, the effect was not significant. Moreover, there was no significant correlation between preoperative weight loss and postoperative complications.

**Conclusion:** In our retrospective study, we were not able to find a significant effect of postoperative complications on patients' weight after six or twelve months. However, there was a distinct tendency towards weight loss after twelve months. Thus, it is likely that a correlation between these two factors exists. So as to gain more information about the problem, prospective trials with a larger number of patients should be conducted.

## L‐leucine nutritional supplementation modulates mTOR cell signalling pathway in the muscle under Walker‐256 tumour growth conditions

Bread Cruz, André Oliveira and Maria Cristina C. Gomes‐Marcondes

*Dept. Structural and Functional Biology, Biology Institute, State University of Campinas, UNICAMP, 13083862, Campinas, São Paulo, Brazil*

Cancer cachexia is characterised by involuntary weight loss associated with systemic inflammation and metabolic changes. Studies aiming to maintain lean body mass in cachectic tumour‐bearing hosts have made a significant contribution to the reduction of deaths and improvements in the quality of life. In recent years, leucine has demonstrated effective action in maintaining lean body mass by decreasing muscle protein degradation. Despite some evidence suggesting that leucine participates in the same pathway as that used for insulin signalling during protein synthesis, the exact mechanism by which signalling in the mTOR complex occurs remains a subject of constant debate. Much of the evidence regarding the mechanism by which amino acids act on the mTOR pathway is from *in vitro* studies. Therefore, there is a need for further study of the actions of leucine *in vivo,* particularly in a cachectic host. This study aims to assess the effects of a leucine‐rich diet on the signalling pathway of protein synthesis in gastrocnemius muscle during a time‐course of tumour evolution. Wistar rats distributed into experimental groups received or not tumour implant and fed a leucine‐rich diet and were euthanised at three different times following the 7th, 14th and 21st days after tumour growth. We analysed the muscle key proteins of mTOR pathway such as RagA GTPase, ERK/MAP4K3, PKB/Akt, mTOR, p70S6K1, Jnk, IRS‐1, STAT3, and STAT6. The results showed that tumour's effects reduce RAG activation associated with a reduction of the IRS‐1, increased PKB/Akt and Erk/MAP4K3 on the 21st day, and maintenance of p70S6K1; these key proteins were associated with an increase in muscle STAT‐3 and STAT‐6 levels in tumour‐bearing rats. Meanwhile, the leucine supplementation modulates key steps of the mTOR pathway by triggering the increased activation of RagA and mTOR, maintaining the levels of JNK, STAT‐3 and STAT‐6 in muscle during tumour development in animals suffering from a cachectic state.

## Adaptive response of the oxidized lipidome in human plasma following severe burn injury

Lisa A. Baer ^1,3^, Adam C. Lehnig^1^, Francis J. May^1^, Hannah E. Rockwell^2^, Michael A. Kiebish^2^, Niven R. Narain^2^, Charles E. Wade^3^ and Kristin I. Stanford^1^

^1^ *The Ohio State University, Columbus, Ohio, USA;* ^2^ *BERG Health, Framingham, MA;* ^3^ *Center for Translational Injury Research, University of Texas Health Science Center‐Houston, Houston, TX, USA*

After severe injury, marked and sustained increases in catecholamines, cytokines and cortisol initiate a cascade of events leading to a hypermetabolic response and ensuing cachexia. This cachexic response is characterized by an increase in energy expenditure and a massive loss of body mass, predominantly a result of increased lipolysis, and is accompanied by an inflammatory response. Recently, circulating lipids called “lipokines” have been identified that can act as secreted signaling molecules to alter whole‐body metabolic health and inflammation, thus raising the possibility that there may be lipokines secreted in response to burn injury that could regulate the hypermetabolic response and inflammatory cascade observed post‐injury. To determine if burn injury alters signaling lipids in humans, we performed liquid chromatography‐mass spectrometry (LC‐MS) on serial plasma samples taken post burn injury at baseline (day 1), early (days 2–4), mid (days 5–9) and late (days 15–30) from 7 consented male and female patients, aged 47 + 7 yrs, with total body surface area burn injury of 67 + 6%, and compared these to normal control samples. The concentration of 108 species of oxidized lipids with annotated signaling function was measured. We detected 8 lipid species whose concentration was significantly increased in the plasma of burn patients in the baseline sample compared to controls. Interestingly, each lipid that was significantly increased is a metabolite of the arachidonic acid (AA) cascade. Of these 8 lipids, 15‐HETE, involved in the inflammatory response, was consistently increased compared to normal subjects throughout the first 7 days post‐burn injury. Notably, its downstream metabolite, 15‐oxoETE, was also significantly increased in the circulation early, mid, and late post‐burn injury. The source of these lipids, as well as their downstream targets, is currently under investigation. These data may assist in identifying potential therapies to benefit burn patient outcome.

## Cancer‐induced cachexia *vs.* chemotherapy‐associated cachexia: two sides of the same coin?

Rafael Barreto^1^, Giorgia Mandili^2^, David L. Waning^3^, Frank A. Witzmann^4^, Franco Novelli^2^, Teresa A. Zimmers^1,5,6^ and Andrea Bonetto ^1,5,6^

^1^ *Department of Surgery, Indiana University School of Medicine, Indianapolis, IN, USA;* ^2^ *Centre for Experimental and Clinical Studies, University of Torino, Italy;* ^3^ *Department of Cellular and Molecular Physiology, Pennsylvania State University, Hershey, PA, USA;* ^4^ *Indiana University Proteomics Core Facility;* ^5^ *Simon Cancer Center, Indiana University School of Medicine, Indianapolis, IN, USA;* ^6^ *IUPUI Center for Cachexia Research, Innovation and Therapy, Indianapolis, IN, USA*

Cachexia represents one of the major complications associated with the occurrence of cancer. We recently showed that chemotherapy itself contributes to the development and sustainment of cachexia. Despite this, it is unclear whether similar molecular mechanisms are responsible for the occurrence of cachexia resulting from either chemotherapy treatment or tumor growth. The purpose of the present study was to investigate the cachexia signature associated with muscle wasting due to chemotherapy or cancer.

The growth of the C26 tumor caused progressive depletion of muscle tissue, along with general inflammation, muscle weakness and changes in mitochondrial morphology. Similarly, administration of Folfiri, a combination of 5‐fluorouracil, leucovorin and CPT‐11, caused loss of muscle mass, decrease in muscle strength and overall depletion in muscle mitochondrial content. By means of a LC/MS quantitative approach, we identified 387 proteins significantly modulated in the muscle of Folfiri‐treated mice, and 269 proteins differentially expressed in the C26 hosts (*p* < 0.05, −1.5 ≥ fold change ≥ +1.5). The pathway analysis revealed mitochondrial dysfunctions in both experimental conditions, consistently with changes in the expression of mediators of mitochondrial dynamics and biogenesis. Alterations of oxidative phosphorylation, TCA cycle, fatty acid metabolism and Ca^2+^ signaling were also detected. Overall, the proteomic signature in the presence of both chemotherapy and cancer suggested the activation of mechanisms associated with movement disorders, necrosis, muscle cell death, muscle weakness and muscle damage and, conversely, was consistent with the inhibition of pathways regulating nucleotide and fatty acid metabolism, synthesis of ATP, muscle and heart function, as well as ROS scavenging. Interestingly, strong up‐regulation of pro‐inflammatory acute‐phase proteins and a more coordinated modulation of mitochondrial and lipidic metabolisms were observed in the muscle of the C26 hosts, differently from the Folfiri‐treated animals.

In conclusion, our results suggest that cancer and chemotherapy contribute to muscle loss by activating several common signaling pathways. Regardless, peculiar differences resulting from the effects due to either cancer or chemotherapy are also shown. In an attempt to translate our findings into the clinical setting, these data support the undertaking of combination strategies that aim to both counteract tumor growth and reduce chemotherapy side effects.

## Reassessing biomarkers in cancer cachexia

Erin E. Talbert, Jennifer Yang, Thomas A. Mace, Matthew R. Farren, Priyani Rajasekera, Ericka Haverick, Heather Lewis, Mark Bloomston, Tanios Bekaii‐Saab, Carl Schmidt, Mary Dilhoff, Phil Hart, Gregory B. Lesinski and Denis C. Guttridge

*Arthur G. James Comprehensive Cancer Center Cancer Cachexia Program, The Ohio State University Medical Center, Columbus, Ohio, 43210, USA*

The current focus of our laboratory remains on understanding the role of the muscle microenvironment as a cause of wasting in cancer cachexia. More recently, we are pursuing several pre‐clinical studies to identify compounds that can be translated to the clinic, as well as identifying biomarkers that might be predictive of cachexia or poor outcomes associated with the cachexia syndrome. Two such studies will be discussed in our presentation.

The first relates to an MEK inhibitor that was utilized in a clinical trial at our institution treating patients with cholangiocarcinoma, which is a tumor type with a high prevalence of cachexia. While only 12% of patients had objective responses to therapy, 80% of patients treated with the MEK inhibitor experienced significant weight gain associated with increases in skeletal muscle mass. These clinical data suggested that MEK inhibition might possess an anti‐cachectic activity. We tested this hypothesis by dosing C‐26 tumor‐bearing mice with the MEK inhibitor, MEK162. Results showed that MEK162 preserved skeletal muscle mass but also revealed a modest effect on tumor growth. To determine whether the anti‐cachectic activity of MEK inhibition could be uncoupled from its tumor effect, we produced a C‐26 tumor line that was resistant to MEK162, which we named C‐26R. Importantly, despite the aggressive nature of C‐26R tumors, treatment with MEK162 remained efficacious in sparing body weight and muscle mass compared to vehicle‐treated mice. Evidence suggests that MEK inhibition prevents atrophy by decreasing autophagic activity.

In a second study, we collected peripheral blood from pancreatic cancer patients undergoing attempted surgical resection of their tumors. Patients were classified as non‐cachectic (*n* = 28) or cachectic (*n* = 50) based on a 5% weight loss criteria. A panel of 25 chemokines/cytokines was screened to determine whether a relationship existed between these plasma biomarkers and pancreatic cancer‐induced cachexia. In chemotherapy naïve patients, cachexia was associated with increases in the chemokine MCP‐1 compared to non‐cachectic patients. The results from the other factors on the panel will be discussed.

## IL‐11 induced cachexia symptoms in cancer via AKT/mTOR signaling in skeletal muscle

Xiangzheng Chen ^1,2^, Yangping Wu^2^, HaiChuan Wang^1^, Mingheng Liao^1^ and YongZeng^1^

^1^ *Department of Liver Surgery and Liver Transplantation Center, West China Hospital, Sichuan University, China;* ^2^ *State Key Laboratory of Biotherapy and Cancer Center/Collaborative Innovation Center for Biotherapy, West China Hospital, West China Medical School, Sichuan University, China*

Cachexia is a complex metabolic disorder syndrome, consumption of skeletal muscle as main feature, in advanced cancer patients with unclear pathogenesis and invalid treatment. IL‐11, a member of IL‐6 family, is clinically applied to treat cancer patients with thrombocytopenia after chemotherapy. In recent years, studies have proved that IL‐11 could promote multiple tumors growth and had a correlation with tumor metastasis and poor prognosis. Studies have demonstrated the close relationship between IL‐6 and cancer‐related cachexia, while it is still unclear that whether IL‐11 played a role in cancer cachexia. Our former experiments have illustrated that IL‐11 was upregulated with the development of mouse tumor cachexia, while AKT/mTOR signal pathways, key regulators for skeletal muscle cell growth, showed a downward trend. This study is to detect serum levels of IL‐11 at each stage of mouse cancer cachexia models as well as AKT/mTOR pathways in skeletal muscle and tumors in correspondence. Furthermore, these results will be validated *in vitro* and among samples of cancer cachexia patients. This study aims to clarify the function of IL‐11 in cancer cachexia and to provide theoretical support for further study on the pathogenesis and treatments to cancer cachexia.

## Exosomes as neuroendocrine modulators in cancer cachexia

Katherine A. Michaelis^1^, Kevin G. Burfeind^1^, Xinxia Zhu^2^ and Daniel L. Marks ^2^

^1^ *MD/PhD Program, Oregon Health & Science University, USA;* ^2^ *Department of Pediatrics, Oregon Health & Science University, USA*

A great deal of work has demonstrated that tumors produce factors that act directly on muscle to induce tissue catabolism. However, our research argues that signaling between tumor and the central nervous system is also critical for the metabolic, behavioral, and systemic complications seen in cancer cachexia, including muscle wasting. The mediobasal hypothalamus (MBH) is uniquely equipped as both a sensor and amplifier of peripheral inflammatory signaling. This region contains both sensory and secretory circumventricular organs (CVOs) with an attenuated and dynamic blood brain barrier that confers the ability to detect peripheral signaling mediators. Inflammatory signaling from the periphery is detected and amplified into a central inflammatory response in the MBH, thereby altering the activity of key neuronal populations regulating the metabolic and behavioral features of cachexia. It is now clear that extracellular vesicles (EVs) containing proteins, lipids, and RNA transmit a great deal of information within the tumor microenvironment. These can perform numerous functions in cancer, including promoting metastasis, chemotherapy resistance, and disease progression. Furthermore, a recent study demonstrated that tumor‐derived exosomes directly induce muscle wasting, suggesting a role in lean body mass loss in cachexia. This talk will present data supporting the idea that extracellular vesicles derived from growing tumors signal as endocrine modulators that drive cachexia via the induction of inflammation within the MBH and subsequent illness behaviors and neuroendocrine dysfunction. Specifically, we demonstrate that EVs obtained from cultures of pancreatic cancer potently induce sickness behaviors, weight loss, and muscle catabolism when delivered centrally *in vivo* and induce a pro‐inflammatory response in cultured microglia *in vitro.* Animals bearing this tumor also demonstrate cachexia associated with hypothalamic inflammation. These data support the primary hypothesis and suggest that endocrine signaling via tumor‐derived EVs represents a novel therapeutic target in cancer cachexia.

## Mechanisms of action of ghrelin in cancer cachexia

Jose M. Garcia

*Geriatric Research, Education and Clinical Center, VA Puget Sound Health Care System, University of Washington School of Medicine, USA*

Ghrelin is a novel anabolic hormone that increases energy intake and decreases energy expenditure and inflammation leading to an increase in muscle and fat mass. Based on these properties, ghrelin is currently one of the pathways most advanced in development as a treatment for cancer anorexia and cachexia. However, its mechanisms of action in the setting of cancer cachexia are not fully understood.

To this date, the only identify receptor for ghrelin is the growth hormone secretagogue receptor 1a (GHSR‐1a or GRLN) and this receptor, although present in the central nervous system, is not present in muscle or adipose tissue. Ghrelin's GH secretagogue and appetite stimulating effects appear to be dependent on the GRLN present in the pituitary gland and the hypothalamus. These central effects are likely to have secondary positive effects on other peripheral targets such as skeletal muscle and adipose tissue. However, recent data suggest that some of ghrelin's effects in these tissues are GRLN independent.

The objectives of this talk will be to review the evidence for the different mechanisms of action being proposed for ghrelin and the GRLN in different target organs in rodents and also to review the information available from clinical trials regarding the mechanisms of action for ghrelin/GRLN agonists in healthy humans and in patients with cancer cachexia.

The development of therapies for the prevention or treatment of cancer‐related fat and muscle wasting is desperately needed. Unraveling the mechanisms mediating ghrelin's protective effects in this setting could allow better targeting of this pathway, and the development of novel therapies for cancer cachexia and for other conditions such as chronic obstructive pulmonary disease, heart failure and frailty of the elderly is also associated with cachexia and anorexia.

## Novel treatment approaches to cancer cachexia: Results from two recent randomized controlled trials using essential amino acids and espindolol

Stephan von Haehling

*Department of Cardiology and Pneumology, Georg‐August‐University, Göttingen, University of Göttingen Medical Centre, Göttingen, Germany*

**Background:** Cancer cachexia occurs frequently among patients with advanced pancreatic, colorectal, or non‐small cell lung cancer. Therapeutic options are limited, and novel treatments to counterbalance the wasting process are urgently needed. Potential treatments embrace anabolics and nutritional supplements.

**Methods:** Two double‐blind, randomized, placebo‐controlled trials were conducted. The first trial (the ACT‐ONE trial) used espindolol, a novel non‐selective beta‐blocker with central 5‐HT1a and partial beta2 receptor agonistic effects. A total of 87 patients with cancer cachexia (colorectal cancer, *n* = 29; non‐small cell lung cancer, *n* = 58) were randomized to placebo or a high (10 mg bd) or a low (2.5 mg bd) dose of espindolol. Body weight, body composition (DEXA), handgrip strength, and exercise capacity were assessed at baseline and after 16 weeks of treatment. The second trial used a nutritional supplement consisting of all essential amino acids. A total of 50 patients (colorectal cancer, *n* = 20; pancreatic cancer, *n* = 30) received 4 g of essential amino acids three times daily (12 g/daily) over 16 weeks. Patients (age: 61.7 ± 10.4 years, 86% stage IV) had their exercise capacity (stair‐climbing power), body composition (dual energy X‐ray absorptiometry, DEXA), quality of life, and muscle strength assessed at baseline and after 8 and 16 weeks.

**Results:** In the first trial, treatment with espindolol was associated with significant increases in body weight, lean mass, and handgrip strength. Trends towards improvement were noted for stair‐climbing power and 6‐minute walk distance with espindolol treatment. In the first trial, only total fat mass increased significantly from baseline to week 16 in the group that received essential amino acids (essential amino acids: +1287 ± 2391 g, placebo: ‐2544 ± 5224 g, *p* = 0.02). No significant change was noted with regard to stair‐climbing power, 6‐minute walk distance, body weight, total or appendicular lean mass, or quality of life.

**Conclusions:** Treatment with espindolol, particularly using the higher dose, was associated with beneficial effects on body weight and functional parameters among patients with cancer cachexia. Larger studies should verify these positive results. Treatment with essential amino acids showed improvement in fat mass but failed to improve lean mass or functional parameters.

## Eccentric contraction regulation of mTORC1 signaling in cachectic mouse skeletal muscle

Justin P. Hardee ^1^, Song Gao^1^, Brandon N. VanderVeen^1^, Dennis K. Fix^1^ and James A. Carson^1,2^

^1^ *Integrative Muscle Biology Laboratory, University of South Carolina, Columbia, SC, USA;* ^2^ *Center for Colon Cancer Research, University of South Carolina, Columbia, SC, USA*

Eccentric contraction (ECC)‐induced skeletal muscle hypertrophy involves the activation of protein synthesis through mTORC1 signaling. While cancer cachexia disruption of muscle protein turnover regulation is well established, the sensitivity of cachectic muscle protein synthesis to ECCs warrants further investigation. We have established that severely cachectic muscle can initiate a growth response to repeated ECCs. However, the role of mTORC1 activation in this growth process was not established. Therefore, we examined the ECC responsiveness of cachectic muscle mTORC1 signaling and protein synthesis using 2 distinct preclinical cancer cachexia models. Male, *Apc^Min/+^* (*N* = 9; 16% body weight loss) and Lewis Lung Carcinoma (LLC) tumor‐bearing (*N* = 8; 14% body weight loss) mice performed a single bout of ECC (10 sets of 6 repetitions), and mTORC1 signaling was examined 3 h post‐contraction. The left tibialis anterior (TA) performed ECC, while the right TA served as an intra‐animal control. Age‐matched C57BL/6 (WT) mice served as controls. In control muscle *Apc^Min/+^* and LLC, mice had decreased muscle mass, p70S6K(T389) phosphorylation, and protein synthesis compared to WT controls. Cachexia did not inhibit the acute ECC‐induction of p70S6K(T389) phosphorylation in *Apc^Min/+^* or LLC mice. While ECC induced protein synthesis in *Apc^Min/+^* mice, protein synthesis remained suppressed compared to WT mice. Interestingly, ECC did not stimulate muscle protein synthesis in LLC mice. Although cachexia suppressed muscle anabolic signaling, cachectic muscle maintained the ability to activate mTORC1 signaling by ECC. These results demonstrate that ECC induced responsiveness of mTORC1 signaling is maintained in cachectic muscle but that there is a disconnection between this responsiveness and the activation of muscle protein synthesis. Supported by NIH/NCI R01‐CA121249.

## Peptide‐mediated maintenance of adipose tissue integrity protects against cancer cachexia

Maria Rohm, Michaela Schäfer, Victor Laurent, Carolyn Algire, Mauricio Berriel Diaz and Stephan Herzig

*Institute for Diabetes and Cancer, Helmholtz Center Munich, 85764 Neuherberg, Germany, and Joint Heidelberg‐IDC Translational Diabetes Program, Inner Medicine 1, Heidelberg University Hospital, 69120, Heidelberg, Germany*

Cancer cachexia represents an energy wasting syndrome in a large number of tumor patients, which significantly contributes to poor quality of life, resistance to chemotherapy and high‐mortality rates. However, the underlying molecular mechanisms are still incompletely understood, which is also reflected by the lack of effective and routine therapeutic measures to counteract cachexia progression. Besides muscle wasting, loss of adipose tissue is increasingly recognized as a central component of cachexia. Interestingly, depletion of white adipose tissue (WAT) as the body's main energy storage organ and fatty acid source generally precedes the loss of skeletal muscle mass upon tumor development.

Here, we show that tumor cell supernatant exposure of white adipocytes as well as tumor growth in mice triggered a futile energy wasting cycle in white adipose tissue (WAT). While uncoupling protein (UCP)1‐dependent thermogenesis was dispensable for tumor‐induced body wasting, cachectic WAT was consistently characterized by the simultaneous induction of both lipolytic and lipogenic pathways and – despite a low cellular energy status – a paradoxically inactivated AMP‐activated protein kinase (AMPK). The observed degradation of AMPK in WAT of cachectic mice was – at least in part – mediated by interaction with cell death‐inducing DNA fragmentation 45‐like effector a (CIDEA), leading to the uncontrolled loss of lipid energy stores.

Indeed, an AMPK stabilizing peptide (ACIP) was able to ameliorate WAT wasting *in vitro* and *in vivo* by shielding the CIDEA‐targeted interaction surface on AMPK, thereby promoting survival of tumor‐bearing animals. Thus, our data establish the remodeling of adipocyte lipid homeostasis as a key event in tumor‐induced WAT wasting and propose the ACIP‐dependent preservation of WAT AMPK integrity as a concept in future cachexia therapies.

## Identification of new tumor‐borne mediators triggering alterations in fat, muscle and cardiac function in cancer cachexia

Victor Laurent, Michaela Schäfer, Mauricio Berriel Diaz and Stephan Herzig

*Institute for Diabetes and Cancer (IDC), Helmholtz Center Munich, Neuherberg, Germany, and Joint Heidelberg‐IDC Translational Diabetes Program, Heidelberg University Hospital, Heidelberg, Germany*

Cancer cachexia affects the majority of patients suffering from advanced cancers. Despite its clinical importance, the identity of tumor‐borne signals and their impact on specific peripheral organ systems remain mostly unknown. By using different mouse models for colorectal cancer‐dependent cachexia, we show here that skeletal muscle and fat tissues mass as well as cardiac muscle mass and function were affected in cancer cachexia and that the cachexia‐induced effects were mediated by tumor‐secreted factors in a cell autonomous manner. In an unbiased differential secretome analysis of colon cancer cells combined with high‐throughput cardiomyocyte phenotyping, we defined a set of tumor‐secreted mediators with cachexia‐inducing capacities. Notably, signature of seven “cachexokines” was sufficient to mediate both, atrophy and aberrant fatty acid metabolism in primary cardiomyocytes. Amongst these seven candidates, Ataxin10 was found to represent a robust cachexia indicator as its serum levels were elevated in both murine and human cancer cachexia. Taken together, our study demonstrates that cardiac dysfunction is an underestimated clinical feature of cancer cachexia and those alterations in fatty acid metabolism represent a distinct feature of the cachectic heart. In addition, this study provides an unbiased and functional screening setup for the investigation of tumor secreted factors with cachexia‐inducing capacity. Finally, further experiments are ongoing to characterize candidates specifically involved in fat and muscle wasting. The identification of new cachexokines will provide a rational approach towards personalized predictive, diagnostic and therapeutic measures in cancer cachexia.

## Therapeutic effects of greater systemic IL‐15 on mammary tumor‐associated muscle fatigue

Joseph Bohlen^1^, Sarah L. McLaughlin^2,3^, Sijin Wen^4^ and Emidio E. Pistilli ^1,3,5^

^1^ *Division of Exercise Physiology;* ^2^ *Animal Models and Imaging Facility;* ^3^ *West Virginia University Cancer Institute;* ^4^ *Department of Biostatistics;* ^5^ *West Virginia Clinical and Translational Sciences Institute, West Virginia University, Morgantown, WV, USA*

The aims of this project were twofold: (1) determine alterations in muscle fatigue properties following mammary tumor growth in mice; (2) determine therapeutic benefit of greater systemic levels of IL‐15 on muscle fatigue properties following mammary tumor growth. We hypothesized that mammary tumor growth would increase the rate of muscle fatigue and that greater systemic levels of IL‐15 would attenuate these alterations in fatigue. C57BL/6 WT mice (*n* = 23) were used to determine the effects of mammary tumor growth on fatigue responses in the type II fiber extensor digitorum longus (EDL) muscle, as type II muscles are typically susceptible to cancer‐induced fatigue. Muscle‐specific IL‐15 over‐expressing mice (*n* = 19) and littermate control mice (*n* = 25) were used to determine therapeutic benefit of greater IL‐15 on muscle fatigue in response to tumor growth. Mammary adenocarcinomas were induced in immunocompetent mice for 2 or 4 weeks using the E0771 cell line. EDL muscles from WT mice fatigued at a greater rate following 4 weeks of tumor growth, compared to muscles from non‐tumor bearing mice and mice following 2 weeks of tumor growth. The area under the fatigue curve, representative of the total amount of force produced during the fatigue protocol, was significantly less in EDL muscles from WT mice following 4 weeks of tumor growth compared to muscles from non‐tumor bearing mice and mice following 2 weeks of tumor growth. This greater fatigue was associated with a lesser mitochondrial DNA content in muscles from WT mice exposed to tumors for 4 weeks. Four weeks of tumor growth induced a greater rate of fatigue in EDL muscles from IL15 over‐expressing mice and littermate control mice, when compared to strain‐specific non‐tumor‐bearing mice. However, despite a loss of muscle mass, the greater area under the fatigue curve in muscles from IL‐15 over‐expressing mice indicates a greater ability to sustain force output, compared to littermate control mice. These data support the hypothesis that while IL‐15‐based therapies may not prevent mammary tumor‐associated muscle loss, IL‐15 therapies may be beneficial for reducing mammary tumor‐associated muscle fatigue. These observations lay the framework for further mechanistic studies of IL‐15‐based therapies in breast cancer‐associated fatigue.

## Systemic inflammation is associated with concurrent inflammatory gene expression in skeletal muscle of advanced cancer patients

Cynthia Stretch, Vera C Mazurak, Sambasivarao Damaraju, Oliver Bathe and Vickie E Baracos

**Background:** Serum C‐reactive protein (CRP) is the most widely accepted index of systemic inflammation. It is shown to have prognostic significance in cancer and is a proposed biomarker of the catabolic drive, which occurs to varying degrees in patients with cancer cachexia. The extent to which skeletal muscle may show localized inflammation in cancer cachexia is unknown.

**Design and method:** Pre‐surgery serum CRP was measured in patients with gastrointestinal cancers at risk for cachexia who underwent open abdominal surgery as part of their clinical care (*n* = 67). *Rectus abdominis* muscle biopsies were taken from the site of incision at the start of surgery. Total muscle RNA was isolated and analyzed for gene expression using Agilent 41 K human arrays.

**Results:** The distribution of CRP values was 7.6 ± 8.0 mg/L. We identified 1097 transcripts correlated (*r* > |0.3|, *p* ≤ 0.01) with serum CRP. Correlated transcripts were evaluated using Ingenuity Pathway Analysis. Fisher's Exact Test identified biological functions associated with correlated transcripts. The top biological functions were associated with inflammatory response and immune cell trafficking (e.g. activation of leukocytes (*p* = 2.18E‐10) and neutrophils (*p* = 3.17E‐7), movement of macrophages (*p* = 2.59E‐6) and accumulation of leukocytes (*p* = 2.75E‐6). Main upstream regulators identified included IL1B (*r* = 0.30; *p* = 0.01), IFNG (*r* = 0.34; *p* = 0.005), VEGFA (*r* = 0.33; *p* = 0.006), CSF2 (*r* = 0.41; *p* = 0.001), IL4 (*r* = 0.34; *p* = 0.004) and PTGS2 (*r* = 0.41; *p* = 0.001). Most of these upstream regulators feed into the NF‐kB complex that acts as a major regulatory centre. One subunit of the NF‐kB complex correlated with CRP (NFKB1 *r* = 0.41; *p* < 0.001). Downstream transcription regulators associated with elevated serum CRP included STAT3 (*r* = 0.34; *p* = 0.007), HIF1 (*r* = 0.31; *p* = 0.01), HMGB1 (*r* = 0.39; *p* = 0.001) and NFATC2 (*r* = 0.40; *p* = 0.001).

**Conclusion:** Muscles of patients with cancer show expression of multiple classes of molecules from inflammatory pathways. Positive correlations between serum CRP and skeletal muscle expression of molecules involved in immune‐cell recruitment/activation/accumulation, and cytokine signaling suggests innate immune response locally within the muscle. These findings are concordant with prior reports that reduced rates of cachexia are observed in patients with a loss of function mutation in P‐selectin, a cell adhesion molecule involved in leukocyte extravasation.

## Are changes in phase angle and body composition by gender in cancer patients with cachexia different from healthy population?

Saunjoo Yoon ^1^, Oliver Grundmann^1,2^, Joseph J. Williams^3^, Thomas. J. George^4^ and Lucio Gordan^5^

^1^ *College of Nursing, University of Florida;* ^2^ *College of Pharmacy, University of Florida;* ^3^ *Sunshine Integrative Health;* ^4^ *Hematology/Oncology, College of Medicine, University of Florida;* ^5^ *Florida Cancer Specialists & Research Institute, Gainesville, FL, USA*

**Significance** & **Background:** Bioelectrical impedance analysis (BIA) is a non‐invasive and inexpensive tool to evaluate body composition in patients with cancer cachexia due to disproportional loss of muscle mass and accumulation of fluid due to inflammation. Body composition and phase angle (PA) correlate with nutritional status and overall survival. The purpose of this study is to investigate gender differences in body composition with a focus on extracellular (ECW) and intracellular water (ICW), fat mass (FM), and PA of gastrointestinal (GI) cancer patients compared to a healthy reference population. We hypothesize that gender differences in body composition in cancer patients do not differ from reference values.

**Methods**, **Intervention Analysis:** The study is a two‐group, single blind, randomized controlled design to examine the effect of an acupuncture intervention on appetite in GI cancer patients with cachexia. Baseline BIA data of 38 subjects (M:F = 21:17) were extracted and compared to reference values taken from the NHANES‐III dataset surveyed in healthy Americans. Participants were included if they lost 5% or more weight over a 6‐month period. Student's *t*‐test with *p* < 0.05 was used for statistical comparisons.

**Findings** & **Interpretation:** Male and female cancer patients did not differ in age. Age‐matched cancer patients did not differ from healthy controls in FM changes. Women had significantly higher FM (*p* = 0.00026). PA was statistically significantly lower in both genders compared to the healthy controls. Female patients had significantly higher reductions in PA compared to males. There was a strong correlation between PA and ICW differences for both genders (female: *r* ^2^ = 0.772, *p* < 0.001; male: *r* ^2^ = 0.951, *p* < 0.001). A statistically significant shift from ICW to ECW in men was observed, whereas the opposite occurred for women relative to their controls.

**Discussion** & **Implication:** Since body weight does not reflect true health status of a cancer patient, BIA should be an integral part to evaluate health. Cachexia affects women and men differently in regard to body composition changes on FM and water shifts. This study indicates PA as a suitable surrogate marker for changes in cell composition and health status. Considering the prevalence of cachexia in cancer patients and limited research on gender‐specific BIA changes in such a population, this research warrants further investigation.

## Drosophila models of cachexia and other cancer‐induced systemic syndromes

David Bilder

*University of California Berkeley, USA*

Malignancy, defined as the ability of a growing tumor to kill its host, lies at the root of the cancer problem. The mechanisms underlying malignant lethality remain mysterious and can benefit from analysis in simple, reductionist model systems. Many features of malignancy are recapitulated in the Œneoplastic tumors of the fruit fly Drosophila, and precedent demonstrates the value of Drosophila studies for human cancer biology, due to conservation of molecular mechanisms. We have found that Drosophila tumors growing in WT adults induce tissue wasting resembling human cancer cachexia, through a mechanism involving JNK‐regulated activation of a systemic insulin antagonist. I will discuss these studies as well as extensions to other tumor–host interactions driving morbidity and mortality, including unbiased genetic screening for new regulators of the host response to malignant tumors.

## Cancer regulation of contraction‐induced anabolic signaling in skeletal muscle

James Carson

*Professor, Division of Applied Physiology, Exercise Science Department, University of South Carolina, Columbia, South Carolina, USA*

The preservation of skeletal muscle mass remains a critical goal for the successful treatment of the cancer patient. To this end, preclinical mouse studies over the past decade have dramatically advanced our mechanistic understanding of cancer‐induced muscle wasting. However, discrepancies have emerged between human cancer patients and preclinical models regarding cellular mechanisms and systemic drivers of the wasting process. These discrepancies may be related to skeletal muscle's ability to respond to environmental stimuli involving loading patterns, daily contractile activity, nutrition, and hormonal stimuli. Preclinical studies often quantify fasted, sick mice, undergoing very little volitional activity. This has created significant gaps in our understanding of cachectic muscle's anabolic response to environmental stimuli. The primary objective of this presentation is to highlight the effect of the cancer environment on mouse muscle's response to anabolic stimuli involving nutrition and muscle contraction. Data will be presented from experiments employing the ApcMin and Lewis Lung Carcinoma (LLC) mouse cachexia models. We will first examine muscle functional changes that occur with the progression of cachexia in tumor bearing mice, and their relationship to systemic and local inflammation. Next, we will examine if the cachectic environment can disrupt the muscle's ability to activate anabolic signaling in response to contractile and nutritional stimuli. Lastly, we will examine if repeated bouts of muscle contraction have the capacity to alter cachexia muscle's anabolic response to either contraction or nutritional stimuli. The presentation will point to the need to better understand the potential synergism between muscle disuse and the cancer‐induced cachectic environment for the acceleration of muscle wasting.

## Targeting protein breakdown to improve cancer‐associated muscle wasting

Paola Costelli

*Department of Clinical and Biological Sciences, University of Turin, Italy*

Alterations of protein metabolism including increased protein breakdown, reduced protein synthesis or both are frequent features of muscle wasting in cancer cachexia. In this regard, results obtained in pre‐clinical models have shown that enhanced protein degradation behaves as the major player, although data on cancer patients are not conclusive so far.

Depending on the experimental model adopted, all the four proteolytic systems operating in the skeletal muscle have been shown to differently contribute to cancer‐induced muscle wasting. In this scenario, however, the most relevant degradative pathways are those dependent on ubiquitin‐proteasome and autophagy. These results paved the way to the design of therapeutic approaches aimed to target overall protein breakdown also by selectively inhibiting a single proteolytic system.

Data available in the literature show that systemic treatments with drug such as β2‐adrenergic agonists or hormones such as insulin or ghrelin are useful to prevent the onset of muscle wasting and protein hypercatabolism in experimental cancer cachexia, although they can exert dangerous side effects. On the other side, specific proteasome or autophagy inhibition did not exert appreciable positive results.

More recently, pre‐clinical therapies have focused on specific molecular pathways, by both pharmacologic and genetic approaches such as transgene overexpression or RNA interference. The data obtained point to the need of therapies able to inhibit overall protein breakdown rather than to modulate a single proteolytic system. In addition, taking into account the multifactorial nature of cancer cachexia, anti‐catabolic strategies should be considered just one component of the therapeutic management of cancer patients.

## NCI clinical trial of testosterone to combat cachexia in advanced cancer

Traver J. Wright, Edgar L. Dillon, William J. Durham, Albert C. Chamberlain, Kathleen M. Randolph, Christopher P. Danesi, Astrid M. Horstman, V. Hoang, Maurice Willis, Gwyn Richardson, Sandra S. Hatch, Daniel C. Jupiter, Randall J. Urban, Susan McCammon* and Melinda Sheffield‐Moore

*The University of Texas Medical Branch, Galveston, TX, USA*

Cancer patients with advanced disease often suffer with the syndrome of cancer cachexia. Symptomatically, patients often present with body wasting involving the loss of both lean and fat mass. Despite the prevalence and severity of this wasting syndrome, effective therapies have yet to be realized. We present data from a recently completed NCI funded clinical trial that examined the safety and efficacy of testosterone to combat cachexia in men and women diagnosed with advanced head and neck or cervical carcinoma. Twenty‐one patients of mixed age, race, and gender were randomized to receive weekly intramuscular injections of 100 mg testosterone enanthate (TE) or placebo (PL) for 7 weeks during the early phase of standard of care (SOC) chemotherapy or concurrent chemoradiation. An intent‐to‐treat study design was employed resulting in a study duration that ranged from 31 to 59 days and averaged 47 days. Safety and efficacy assessments were made on the following primary and secondary outcomes: body composition, hormones, blood chemistries, skeletal muscle function, average daily energy intake, resting energy expenditure (REE), duration and intensity of daily activity, quality of life (QOL), and overall 1 year survival. Weekly TE increased total testosterone and free testosterone levels above pre‐treatment values in both men and women in the TE group, with no change in PL. No safety issues were noted at the dose of TE used in either group (TE vs PL) or sex (M vs F), and males and females responded similarly to TE. Over the duration of the study, mean REE decreased on average 9% in PL (*p* = 0.111) and was unchanged in TE (*p* = 0.996). Average daily energy intake (PL: 1374.45 ± 440.90 kcal/day vs TE: 1345.42 ± 387.20 kcal/day) did not differ between PL and TE. The primary outcome of body composition showed that over the duration of the study, PL lost on average 2.0 kg of lean tissue (*p* = 0.085), while TE gained 1.3 kg of lean mass (*p* = 0.062). Although the loss of lean mass in the PL group (3.3%) and the gain in the TE group (3.2%) did not reach statistical significance, the difference in the percent change between groups was significant (*p* = 0.015). PL lost on average 2.9 kg of fat (*p* < 0.001), while TE lost 1.6 kg (*p* = 0.004). Although the mean percent fat loss in PL (17.1%) tended to be greater than in TE (10.7%), this difference did not reach statistical significance (*p* = 0.092). Skeletal muscle peak torque decreased slightly in PL and increased in TE compared to pre‐treatment values. Peak power decreased slightly in both groups (*p* > 0.05). Total scores in the short physical performance battery (SPPB) increased 0.25 points in PL and 1.4 points in TE over the course of the study, although the only test to reach statistical significance in the battery in response to TE was the chair rise score (*p* = 0.033). TE ameliorated the trend for patients to spend a greater portion of the day in sedentary activities and significantly preserved the time TE patients spent participating in lifestyle activities. The social/family well‐being subtest was the most improved on the FACT‐G QOL questionnaire in the TE group and highlighted questions that examine the subjects' subjective interpretation of other's behaviors towards them. Kaplan–Meier survival curves were not different between the two groups (*p* = 0.96) with a one‐year post‐study survival of 55% for TE and 58% PL. Overall, the results of our clinical trial demonstrate that testosterone administration in the early phase of chemotherapy or chemoradiation is safe and efficacious at preserving lean mass in cancer patients with advanced disease undergoing standard of care treatment for cervical or head and neck cancer.

## Distinct, non‐overlapping roles of Gli1 and Gli2 in the molecular regulation of myogenesis

Ernie D Au ^1,2,4^ and Teresa A. Zimmers^1,2,3,4,5^

^1^ *Department of Surgery, Indiana University, Indianapolis, IN, USA;* ^2^ *Department of Biochemistry & Molecular Biology, Indiana University, Indianapolis, IN, USA;* ^3^ *Department of Anatomy and Cell Biology, Indiana University, Indianapolis, IN, USA;* ^4^ *IU Simon Cancer Center, Indiana University, Indianapolis, IN, USA;* ^5^ *IUPUI Center for Cachexia Research Innovation and Therapy, Indiana University, Indianapolis, IN, USA*

Skeletal muscle atrophy can occur with inactivity, malnutrition, or in chronic diseases such as cancer, organ failure, and AIDS. Muscle wasting reduces quality of life, decreases response to therapy, and contributes directly to mortality. Our lab has substantial data implicating the Hedgehog (Hh) pathway as a causal factor in muscle wasting. However, the mechanisms by which the Hh pathway regulates muscle homeostasis have yet to be determined.

*In vitro* data using C2C12 myotubes showed that Hh pathway agonists induced myotube wasting, while antagonists resulted in hypertrophy. Inhibition of the pathway also resulted in an increase in the number of nuclei per fiber, while activating the pathway had the opposite effects. Further investigation into the molecular regulation of myogenesis by the Hh pathway suggests that the Gli family of transcription factors may be key regulators. Analysis of C2C12 myogenesis revealed that the Hh pathway is dynamically regulated and that Gli1 and Gli2 may perform distinct and non‐redundant functions. During myogenesis, we found that mRNA expression levels of *Gli1* are decreased upon differentiation, while expression of *Gli2* is markedly increased. This suggests that Gli1 and Gli2 may exert their effects on separate cell populations, with Gli1 acting in myoblasts and Gli2 in myotubes. Using siRNAs, we found that *Gli1* knockdown significantly increased myosin heavy chain (MHC) expression when compared to a control siRNA. A trend towards larger myotubes with more nuclei per fiber was also observed. Conversely, *Gli2* knockdown resulted in significantly reduced MHC expression and nuclei per fiber, with a trend towards smaller myotubes. Time course analysis of C2C12 cells, knocked down for *Gli2,* undergoing myogenesis revealed a decrease in expression of myogenic regulatory factors (MRFs). Compared to the control, *Gli2* knockdown cells showed decreased expression of *MyoD* and *Myogenin* prior to and through all four days of differentiation. Moreover, *Gli2* knockdown resulted in elevated *Pax7* expression. This increase began upon induction of differentiation and was sustained throughout. Taken together, these data suggest that Gli1 and Gli2 have opposing effects in myogenesis, with Gli1 promoting myoblast/satellite cell proliferation and preventing differentiation, and Gli2 promoting differentiation and expression of MRFs.

## Hypoglycemia during Cachexia is Independent of Hyperinsulinemia

Aditi Narsale, Rosa Moya and Joanna D. Davies

*San Diego Biomedical Research Institute, 10865 Road to the Cure, San Diego, CA, USA*

It is well established that the mechanisms that lead to cachexia in mice carrying either Colon‐26 (C‐26) or Lewis Lung Carcinoma (LLC) tumors are overlapping but not identical. Autophagy is significantly more evident in the C‐26 model than it is in mice with LLC and is caused, at least in part, by hypoglycemia. This study was designed to determine whether C‐26 modulates the regulation of insulin secretion and pancreatic lipase (Pnlip), factors that can modify, and are themselves affected by glucose levels. Two alternative hypotheses are tested: (i) C‐26 induces a higher than normal level of insulin production and secretion by the pancreas causing hypoglycemia, and (ii) C‐26‐induced hypoglycemia is associated with reduced expression of glucose sensitive genes that are critical for insulin and Pnlip production. Thus, lower insulin levels might exacerbate cachexia by reducing the anabolic pathway, while the reduction in triglyceride absorption caused by a reduction in Pnlip might further reduce glucose levels, exacerbating autophagy. We found that pancreata from mice with C‐26‐induced cachexia have a significant reduction in gene expression related to (i) glucose‐sensitive pathways that promote insulin secretion and beta‐cell mass, including a reduction in expression of glucokinase, glucagon‐like peptide 1 receptor (GLP1R), c‐Met, and cell proliferation (Ki67 and PCNA), (ii) both Smad2 and Smad3, part of the TGF‐beta signaling pathway required to maintain beta‐cell function, and (iii) Pnlip. The pancreas is not sensitive to autophagy as indicated by a significant reduction in Beclin‐1 and p62, as well as TRAF6 and Fn14. Moreover, a significant reduction in Akt and mTOR expression indicates an overall reduction in protein synthesis. In contrast, no such changes were evident in the pancreata of mice with LLC tumors. As expected, Atrogin and MuRF1 expression are elevated in both C‐26 and LLC models, and the autophagy pathway (Beclin‐1, Cathepsin‐L, LC3B and p62 expression) is highly active in C‐26 cachexia but less so in LLC cachexia. The data overall are consistent with the notion that cachexia in mice with C‐26, but not LLC, might be exacerbated by a deficiency in insulin secretion and Pnlip, causing a reduction in insulin‐mediated anabolism and enhanced autophagy.

## A comparison of muscle wasting and synthesis signaling between a Type I Diabetes mouse model and a model of cancer‐related cachexia

Yaqi Zhang ^1,2†^, So‐youn Kim^1,2^, Megan M Romero^1,2^, Kelly A Whelan^1,2^, Takehsi Kurita^1,2^, Teresa K Woodruff^1,2^ and Jie Zhu^1*†^

^1^ *Department of Obstetrics and Gynecology, Feinberg School of Medicine, Northwestern University, Chicago, IL, 60611, USA;* ^2^ *Department of Molecular Virology, Immunology & Medical Genetics, The Comprehensive Cancer Center, Ohio State University, Columbus, OH*

*corresponding author

† equal contribution

**Background:** Muscle loss results from an imbalance between anabolic and catabolic processes. In type I diabetes, skeletal muscle wasting is due to nutritional stress; however, muscle loss in the cancer‐related cachexia is not only caused by nutritional stress but also due to the inflammatory cytokines produced by tumor–host interaction. The ubiquitin‐proteasome system plays a key role in muscle wasting in many disease settings, and genes in this pathway are known as atrogenes, including FOXO, which increase in the muscle of both type I diabetes models and cancer‐related cachexia. However, it is still controversial whether FOXO is necessary for the atrogenes pathway, whether the muscle regeneration factors such as Pax7, MyoD, and Myogenin are downstream targets, and whether the TGF beta family members such as GDF8, activin A and activin B regulate the atrogene pathway in different muscle wasting models. In this study, we generated a cachexia and diabetes mouse models. Using these two muscle wasting models, we analyzed the similarities and differences in muscle regeneration, protein synthesis and degradation.

**Methods:** Two muscle wasting models were generated (1) streptozotocin‐induced type I diabetes mice (diabetes mice) and (2) cancer cachexia model using constitutive activation of PI3K in oocyte inducing ovarian granulosa cell tumors (PI3K model). The Tibialis anterior (TA) muscle was isolated from mice models for reverse‐transcription real‐time PCR to detect βA, βB, GDF 8, Atrogin‐1, MuRF‐1, Pax7, Myogenin, MyoD, FOXO3 expression, and detecting protein level by Western Blot for phospho‐Smad2/3, phospho‐FOXO3a, total FOXO3 and phospho‐Akt.

**Results:** (1) βB mRNA, not βA or GDF8, increased in both muscle wasting models. (2) Atrogin‐1 mRNA, not MuRF1, increased in both models. (3) Pax7, but not MyoD1, significantly decreased in both models. (4) Myogenin expression significantly increased in PI3K model but not in diabetes model. (5) Total FOXO3 expression was elevated (measured by mRNA and protein levels) in diabetes model, but not PI3K model; while pFOXO3 increased in PI3K model but decreased in diabetes model, which was confirmed by pAkt protein expression detected by western blot.

**Conclusion:** Nutritional stress and cytokines regulate satellite cell activation and contribute muscle wasting. However, in the degradation pathway, nutritional stress is through FOXO regulation, while the cytokine‐based cachexia signaling pathway bypasses FOXO to trigger ubiquitin‐proteasome system. Importantly, our results point toward activin B as the novel common agonist in muscle wasting.

## BRD4 blockade prevents skeletal muscle loss during cancer cachexia

Marco Segatto^1^, Raffaella Fittipaldi^1^, Hossein Zare^2^, Roberta Sartori^3^, Claudio Fenizia^1^, Cosimo Sperti^4^, Stefano Merigliano^4^, Paola Costelli^5^, Marco Sandri^3^, Panagis Filippakopoulos^6^, Vittorio Sartorelli^2^ and Giuseppina Caretti ^1^

^1^ *Department of Biosciences, University of Milano, Via Celoria 2620133, Milan, Italy;* ^2^ *Laboratory of Muscle Stem Cells and Gene Regulation, NIH/NIAMS, 50 South Drive, Bethesda, MD, USA;* ^3^ *Venetian Institute of Molecular Medicine, 35131, Padova, Italy;* ^4^ *Department of Surgery, Oncology and Gastroenterology, 3rd Surgical Clinic, University of Padua, Italy;* ^5^ *Department of Clinical and Biological Sciences, Unit of General and Clinical Pathology, University of Turin, Italy;* ^6^ *Structural Genomics Consortium, Old Road Campus Research Building, Nuffield Department of Medicine, Oxford, OX3 7DQ, UK*

Skeletal muscle wasting is a hallmark of cancer cachexia, which leads to increased morbidity and mortality, decreased beneficial effects from chemotherapeutic treatment, and poorer quality of life. Therefore, the development of therapeutic avenues addressed at preventing muscle wasting during cancer cachexia is attracting increasing clinical interest. To date, no effective therapies for cachexia are available. Recently, we described that the bromodomain protein BRD4 regulates pro‐atrophic genes and that treatment with the small bromodomain inhibitor JQ1 enhances muscle fiber size, protecting from dexamethasone‐induced muscle atrophy in C2C12 myotubes. In the present study, we evaluated the involvement of BRD4 in skeletal muscle wasting of cachectic mice. To this aim, C26‐tumor bearing mice were chronically treated with the BET inhibitor JQ1 or vehicle. Body weight, skeletal muscle weight and the anabolic/catabolic pathways involved in skeletal muscle homeostasis were analyzed. Our results show that JQ1 treatment blocks muscle‐specific ubiquitin ligases expression and protects tumor‐bearing mice from body weight loss and muscle wasting. Furthermore, JQ1 administration prevents adipose tissue loss and significantly prolongs survival. We show that the BET protein BRD4 promotes cachexia through activation of the muscle atrophy program and through an IL6‐dependent signaling pathway that modulates atrogenes transcription. Consistently, BET proteins pharmacological blockade reduces IL6 systemic levels and prevents the activation of skeletal muscle catabolic genes. Overall, our data uncover that BET proteins may represent a promising therapeutic target to counteract cancer cachexia.

## A novel murine model of pancreatic cancer cachexia: characteristics and advantages of syngeneic Kras^G12D^ P53^R172H^ Pdx‐Cre (KPC) allografts in C57Bl/6 mice

Katherine A. Michaelis ^1^, Xinxia Zhu^2^, Kevin G. Burfeind^1^, Peter Levasseur^2^, Terry K. Morgan^3^ and Daniel L. Marks^2^

^1^ *MD/PhD Program, Oregon Health & Science University;* ^2^ *Papé Family Pediatric Research Institute, Oregon Health & Science University;* ^3^ *Department of Pathology, Oregon Health & Science University, USA*

**Background:** Cachexia is a complex metabolic and behavioral syndrome lacking effective therapies. To address this clinical need, it is essential to have robust preclinical models that accurately recapitulate the multisystemic nature of cachexia. Though Lewis lung carcinoma is often used to induce cachexia in rodents, this model is marked with substantial heterogeneity.^1^ In addition, cachexia remains the deadliest and has the highest prevalence in the context of pancreatic ductal adenocarcinoma (PDAC), in which approximately 80% of patients suffer from the syndrome. We therefore designed an alternate allograft system to study PDAC‐induced cachexia in a preclinical murine model, using a pancreatic tumor cell line from a congenic C57Bl/6 KRAS^G12D^ P53^R172H^ Pdx‐Cre (KPC) mouse.^2^

**Methods:** C57Bl/6 mice aged 7–12 weeks were inoculated subcutaneously, intraperitoneally, or orthotopically with KPC tumor cells. Animal food intake and body weight were monitored daily, with body composition analysis via NMR. Upon development of cachexia, mice were euthanized, and tumor, brain, muscle, liver, blood, and brown adipose tissue (BAT) were harvested. Tumor sections were analyzed histologically. Catabolism genes in muscle, inflammatory response genes in the brain and liver, and thermogenic genes in BAT were compared to tumor naïve controls by qRT‐PCR.

**Results:** All routes of administration produced rapidly growing tumors histologically consistent with poorly differentiated PDAC, featuring desmoplastic stroma and inflammatory infiltrate. KPC tumor growth decreased food intake despite overall increases in body weight (*p* < 0.05), decreased adiposity and lean body mass (*p* < 0.05), decreased muscle weight (*p* < 0.01), and induced skeletal muscle catabolic gene expression of the atrophy‐related ubiquitin ligases MuRF1 and MAFbx (*p* < 0.01)*.* The wasting syndrome in this model was accompanied by increases (*p* < 0.05) in hypothalamic expression of IL‐1β, IL‐1R, and TNFα, and increased hepatic expression of acute phase reactant ORM1. Analysis of locomotor activity, hematologic and endocrine changes, and further characterization of neuroinflammation is ongoing.

**Discussion:** KPC allografts are a robust model for studying cachexia that recapitulate key features of the PDAC disease process and induce a wide array of cachexia manifestations, including muscle wasting, hypothalamic inflammation, and anorexia. This model is therefore well suited for future studies exploring the physiological systems involved in cachexia and for preclinical studies of novel therapies.

**References:**

1. Kir S , White JP , Kleiner S , Kazak L , Cohen P , Baracos VE , Spiegelman BM . Tumour‐derived PTHrelated protein triggers adipose tissue browning and cancer cachexia. Nature. 2014;513(7516):100–4.2504305310.1038/nature13528PMC4224962

2. Westphalen CB , Olive KP . Genetically engineered mouse models of pancreatic cancer. Cancer J. 2012 18(6): 502–510.2318783610.1097/PPO.0b013e31827ab4c4PMC3594661

## The heart and cancer cachexia

Jochen Springer

*Institute of Innovative Clinical Trials, Department of Cardiology and Pneumology, University Medical Centre Göttingen, Göttingen, Germany*

Cardiovascular problems in cancer (cachexia) are usually associated with cardiotoxic effect of chemo‐ and/or radiotherapy. However, there are a number of pre‐clinical studies that show a progressive deterioration of cardiac function in rodent models of cancer cachexia, independently of any anti‐neoplastic therapy. Interestingly, early use of cardiovascular drugs increases the survival rate, while reducing the loss of body weight in a rat model of cancer cachexia. Using colon‐26 cells, a number of secreted mediators were identified that cause atrophy in the myocardium, which was termed cachectokines by the authors, ataxin being the most prominent one. These cachectokines were also found in the colon‐26 mouse model of cancer. In the clinical context, a significant reduction of cardiovascular performance, particularly seen in the heart rate variability, has been described for chemotherapy naïve patients suffering from colon cancer. Therefore, it seems likely that the heart may be affected by cancer independently of anti‐neoplastic therapy making an earlier co‐therapy with cardiovascular medication an interesting option for cancer patients.

## Tumor derived IL6 promotes skeletal muscle atrophy and cachexia in pancreatic ductal adenocarcinoma

Joseph E Rupert ^1,2^ and Teresa A Zimmers^1,2,3,4^

^1^ *Department of Surgery, Indiana University School of Medicine, Indianapolis, IN, USA;* ^2^ *Department of Biochemistry and Molecular Biology, Indiana University School of Medicine, Indianapolis, IN, USA;* ^3^ *Department of Anatomy and Cell Biology, Indiana University School of Medicine, Indianapolis, IN, USA;* ^4^ *Simon Cancer Center, Indiana University School of Medicine, Indianapolis, IN, USA*

Cachexia, or weight loss in cancer, increases morbidity and mortality. In pancreatic ductal adenocarcinoma (PDAC), >85% of patients suffer weight loss with many dying of cachexia. Interleukin‐6 (IL6) is increased in the blood of patients with PDAC and correlates with weight loss and mortality. PDAC tumors often stain heavily for IL6 in stromal cells, with a subset exhibiting staining within tumor cells. IL6 is part of the IL6 family of cytokines, which activates signal transduction through three primary pathways including STAT3, ERK, and PI3K/Akt. This study aims to investigate the role of tumor‐derived IL6 and subsequent activation of the STAT3 pathway in PDAC cachexia.

We developed a murine model of PDAC cachexia using orthotopic injection of tumor cells isolated from the genetic PDAC model LSL‐**K**rasG12D:LSL‐Tr**p**53R172H:Pdx1‐**C**re (KPC). Tumors derived from KPC cells induce muscle wasting and increase cachexia associated with increased IL6 expression. To determine the roles of tumor‐derived IL6 in PDAC cachexia, the *Il6* gene in KPC cells was mutated using CRISPR/Cas9 inducing loss of expression. DNA sequencing of the CRISPR target site verified mutagenesis. QPCR verified substantial reduction in *Il6* mRNA expression (>80% decrease) in the KPC‐IL6 Mutant (IL6 mut) clone.

*In vivo* results showed animals injected with normal KPC cells (KPC) versus those injected with IL6 mut cells had a significantly higher average loss of fat (KPC: −22.3 ± 0.16, IL6 mut: −15.06 ± 0.13%, respectively, *p* < 0.001), and lean tissue (−3.71 ± 0.27, +1.19 ± 0.16%, respectively, *p* < 0.001) measured with Echo MRI. Additionally, KPC injected animals had a significant loss of average muscle mass versus IL6 mut injected animals (Gastrocnemius: −37.9 ± 1.01, −2.8 ± 2.06% and Quadriceps: −19.6 ± 2.1, −3.6 ± 2.09%, respectively, *p* < 0.001). Average IL6 serum levels were significantly higher in KPC versus the IL6 mut injected animals as measured with ELISA (282.07 ± 25.63, 37.17 ± 2.1 pg/ml, respectively, *p* < 0.001). Phosphorylation of STAT3 in the quadriceps muscle of KPC injected animals appeared increased versus IL6 mut injected animals with western blotting. Moreover, tumor‐derived IL6 could be required for tumor growth as the KPC group had significantly larger normalized tumor mass compared to the IL6 mut group (0.04; 0.01, respectively, *p* < 0.01). These results suggest a reduction in the cachexiogenic potential of IL6 mut tumor cells.

## Changes of inflammatory factors in the colon of patients with cancer cachexia

Raquel G Figuerêdo ^1^, Emidio M Matos‐Neto^1^, Joanna DCC Lima^1^, Katrin Radloff^1^, P Leme^1^, Rodolfo G Camargo^1^, Michele J Alves^1^, Estefania Simoes^1^, Ana FM Pessoa^1^, Paulo SM Alcântara^2^, José P Otoch^2^, Alessandro Laviano^3^, Maurizio Muscaritoli^3^ and Marilia Seelaender^1^

^1^ *Cancer Metabolism Research Group, Institute of Biomedical Sciences, University of São Paulo (USP), Av. Lineu Prestes, 1524, 05508‐900, São Paulo, Brazil;* ^2^ *Department of Surgery Clinical, University Hospital, University of São Paulo, São Paulo, Brazil;* ^3^ *Department of Clinical Medicine, Sapienza University of Rome, Italy*

Cachexia is a multifactorial and multiorgan syndrome associated with cancer and other chronic diseases and characterized by severe involuntary loss of body weight, disrupted metabolism, altered immune function, anorexia, fatigue and diminished quality of life. Cachexia affects around 50% of patients with colon cancer and is directly responsible for the death of at least 20% of all cancer patients. Systemic inflammation, the result of the production of inflammatory mediators, is commonly observed in cancer cachexia. It is possible that inflammation may partly derive from the failure in gut barrier function, yielding persistent immune activation. Thus, we investigated colon inflammation in cachexia, performing morphological analysis by light microscopy and quantification of inflammatory factors by Luminex® xMAP in rectosigmoid colon biopsies (20 cm distant from the tumour site) obtained from cachectic (CC = 8) and weight stable (WSC = 6) colon cancer patients, during surgery (ethical approval: CEP‐HU/USP 1385/14). The density of lymphocytic aggregates in the mucosa (*p* = 0.016) was higher in CC than WSC (WSC = 6; CC = 6). The number of eosinophils (*p* < 0.0001) and fibroblasts (*p* < 0.0001) was higher in CC (analysis of 10 fields per slide; WSC = 6; CC = 6). In regard to inflammatory cytokines, interleukin 7 (IL‐7), interleukin 13 (IL‐13) and transforming growth factor beta 3 (TGF‐β3) protein expression was significantly increased in CC, compared with WSC (*p* = 0.02; *p* = 0.048; *p* = 0.048, respectively), and there was a trend towards a higher content of G‐CSF in CC, in relation to WSC (*p* = 0.061) (WSC = 5; CC = 8). The results suggest that there is increased recruitment of immune cells to the colonic mucosa in CC as compared with WSC. Fibrosis was observed, along increased number of eosinophils and fibroblasts in the lamina propria of CC samples as well as higher content of IL‐13 and TGF‐β3. These alterations may suggest gut barrier dysfunction in cancer cachexia. Understanding the role of the gut in the pathogenesis of this syndrome may lead to the development of new therapeutic targets in order to ameliorate intestinal inflammation and the symptoms of cachexia. Sponsored by FAPESP‐12500790.

## Combined HDAC inhibition (AR‐42) and selective androgen receptor modulator (Enobosarm, GTx‐024) administration as cancer‐related cachexia therapy

YC Tseng^1^, Sophia G Liva ^2^, Sally E Henderson^1^, Yi‐Chu Ku^1^, Jason A Benedict^4^, Christopher C Coss^2^, Samuel K Kulp^1^, Tanios Bekaii‐Saab^3^ and Ching‐Shih Chen^1,5^

^1^ *Divisions of Medicinal Chemistry & Pharmacology;* ^2^ *Pharmaceutics & Pharmaceutical Chemistry, College of Pharmacy;* ^3^ *Division of Medical Oncology, Department of Internal Medicine, College of Medicine;* ^4^ *Center for Biostatistics, OSU Comprehensive Cancer Center, The Ohio State University, Columbus, OH, USA;* ^5^ *Institute of Biological Chemistry, Academia Sinica, Taipei, Taiwan*

Cancer‐related cachexia is a complex and debilitating syndrome associated with diminished quality of life, reduced tolerance and response to therapy, and poor clinical outcomes. No effective therapies for cancer cachexia currently exist. We recently reported the effectiveness of the novel class I/IIB histone deacetylase (HDAC) inhibitor AR‐42 (Arno Therapeutics, Inc.) in the C‐26 model of cancer cachexia, in which attenuation of cachexia‐induced loss of skeletal muscle was associated with the suppression of procachexia drivers, including IL‐6, FoxO1, and the E3 ligases, atrogin‐1 and MuRF1. Based on their distinct mechanisms of action, we hypothesized that combining HDAC inhibition with an anabolic agent would provide improved efficacy over either agent alone. Non‐steroidal, orally available selective androgen receptor modulators (SARMs) have recently been developed that demonstrate anabolic activity in skeletal muscle, with greatly reduced undesirable side effects associated traditional steroidal androgen administration. The effects of the SARM, GTx‐024 (enobosarm; GTx, Inc.) and AR‐42, each alone and in combination, were evaluated in the C‐26 model.

Our results show that a fully anabolic dose of GTx‐024 (15 mg/kg, PO, QD) as monotherapy was ineffective in preserving body weight and hind limb muscle mass in C‐26 tumor‐bearing mice. However, when GTx‐024 was combined with a minimally effective dose of AR‐42 (10 mg/kg, PO, QD), attenuation of cachexia‐induced losses in body weight and skeletal muscle mass were apparent and the reductions following combination treatment were significantly greater than those for each agent alone. To verify the androgen dependence of the combination's efficacy, AR‐42 was additionally combined with anabolic doses of dihydrotestosterone (DHT; 3 mg/kg, SC, QD) and the SARM TFM‐4AS‐1 (10 mg/kg, PO, QD) resulting in protective effects similar to those seen with GTx‐024. Consistent with protective effects on muscle mass, hand‐grip dynamometry demonstrated improved muscle function following combined AR‐42 and SARM treatment. These results support further investigation of AR‐42/SARM combination therapy, including mechanistic studies and dose schedule optimization. Given the ongoing clinical evaluation of AR‐42 and several SARMs, our findings are readily translated to the clinic.

## The role of circulating hormones in cachectic colorectal cancer patients: Insulin, C‐peptide, Amylin & Gastric Inhibitory Polypeptide

Estefanía Simoes Fernández^1,2^, Joanna Darck Carola Correia Lima^1^, Raquel Galvão Figueredo^1^, Emidio Marques de Matos‐ Neto^1^, Katrin Radloff^1^, Ariene Murari Soares de Pinho^1,4^, José Pinhata Otoch^1,3,4^, Paulo Sergio Martins de Alcantara^3^, Sílvia Busquets Rius ^5^ and Marília Seelaender^1,4^

^1^ *Cancer Metabolism Research Group, University of São Paulo, Brazil;* ^2^ *Department of pathological anatomy, University Hospital, University of São Paulo, Brazil;* ^3^ *Department of Clinical Surgery, University Hospital, University of São Paulo, Brazil;* ^4^ *Faculdade de Medicina, University of São Paulo, Brazil;* ^5^ *Department of Biochemistry & Molecular Biology, University of Barcelona, Spain*

**Background:** Cancer Cachexia is a multifactorial syndrome characterised by marked weight loss related to skeletal muscle wasting, with or without adipose tissue atrophy. The main symptoms presented are anorexia, fatigue, asthenia, anaemia, insulin resistance and systemic inflammation. Furthermore, this syndrome is directly correlated with death in up to 40% of patients and markedly compromises the effects of pharmacological treatment. Moreover, concentration and secretion of appetite‐regulatory hormone has been proposed to be modified, probably contributing to weight loss and poor prognosis.

**Aim:** To examine circulating appetite‐regulatory hormones and to elucidate possible modified pathways related to cachexia in cancer patients.

**Methods:** Patients with colorectal cancer were divided into Weight‐Stable Cancer (WSC, *n* = 20), and Cachectic Cancer (CC, *n* = 24) groups. Blood sampling was performed after signature of the informed consent form. Hormones and inflammatory markers were quantified with Luminex®xMAP technology.

**Results:** Serum samples from cachectic patients presented a significant reduction of Insulin and some closely related hormones such as C‐peptide, Amylin and Gastric Inhibitory Polypeptide (GIP) when compared to WSC. The serum concentrations were evaluated for Insulin (WSC: 2325 ± 154.6; CC: 1652 ± 62.40, *p* < 0.0003); C‐peptide (WSC: 2979 ± 389.8; CC: 2020 ± 204.2, *p* < 0.0117); Amylin (WSC: 338.7 ± 33.65; CC: 248.7 ± 17.68, *p* < 0.0129) and GIP (WSC: 262.1 ± 47.43; CC: 86.04 ± 13.23, *p* < 0.0002), all of which were lower in cachexia than in weight stable patients. Moreover, positive correlations were found in regard to Insulin/C‐peptide (*p* = 0.009; *r* = 0.6346); Insulin/Amylin (*p* = 0.02; *r* = 0.4717) and Insulin/GIP (*p* = 0.0446; *r* = 0.4225).

**Conclusions:** The results indicate that circulating appetite regulatory hormone concentration is altered in cachectic patients, yet it is not clear whether this represents an effect of the syndrome or a possible cause to the establishment of cachexia‐related alterations. The results point out to a possible beneficial effect of hormone therapy.

## TIR‐domain‐containing adapter‐inducing interferon‐β (TRIF) signaling is important in acute illness response and cancer cachexia

Kevin G. Burfeind ^1^, Xinxia Zhu^2^, Katherine A. Michaelis^1^ and Daniel L. Marks^2^

^1^ *MD/PhD Program, Oregon Health & Science University;* ^2^ *Department of Pediatric Neuroscience, Oregon Health & Science University, USA*

**Background:** Inflammation is a key component of acute illness response and cachexia. In the central nervous system (CNS), hypothalamic inflammation leads to aberrant activity of weight‐ and activity‐modulating neurons, causing the behavioral and metabolic components of cachexia [1]. Mechanisms of inflammatory signaling in the CNS in cachexia remain unclear. Recent data suggest that the MyD88‐independent adaptor TRIF links Toll‐like receptors to cellular activation within the brain [2]. TRIF knockout (TRIFKO) mice are partially resistant to endotoxin‐induced mortality [3]. There are no data demonstrating the role of TRIF in acute illness response or cachexia. We therefore assessed the role of TRIF signaling after lipopolysaccharide (LPS) challenge and in a model of pancreatic cancer cachexia.

**Methods:** WT and TRIFKO mice were challenged with either intraperitoneal (IP) or intracerebroventricular (ICV) LPS. Food intake and body weight were measured; then, hypothalamic gene expression of inflammatory cytokines was measured using qRT‐PCR. Next, WT and TRIFKO mice were inoculated IP with pancreatic ductal adenocarcinoma cells. Food intake and body weight were measured daily. Upon development of cachexia, mice were euthanized, and brain and muscle tissues were harvested. Proteolysis genes in muscle and inflammatory cytokine genes in the brain were measured with qRT‐PCR.

**Results:** TRIFKO mice were resistant to IP and ICV LPS‐induced anorexia and weight loss. TRIFKO mice showed attenuated upregulation of IL‐6 in the hypothalamus after IP LPS (*p* < .001) and attenuated upregulation of P‐selectin, CXCL10, CCL2, and LIF after ICV LPS compared to WT (all *p* < .001). WT mice showed increased serum corticosterone 4 hours after IP LPS injection, while TRIFKO mice did not show a significant increase (315.2 *vs.* 154.8 ng/ml, *p* < .001). TRIFKO mice developed attenuated anorexia compared to WT mice in pancreatic cancer cachexia (91% *vs.* 59% sham food intake over final 5 days, respectively, *p* = .03). Analysis of muscle mass, proteolytic gene expression in muscle, and cytokine gene expression in the brain is ongoing.

**Discussion:** TRIF is an important inflammatory signaling mediator in acute illness response and cachexia. Further studies are needed to investigate the key cell type and receptors involved in TRIF signaling during illness response.

**References:**

1. Braun TP , Marks DL . Pathophysiology and treatment of inflammatory anorexia in chronic disease. Journal of Cachexia, Sarcopenia and Muscle. 2010;1:135–45.10.1007/s13539-010-0015-1PMC306065521475703

2. Yamamoto M , Sato S , Hemmi H , Hoshino K , Kaisho T , Sanjo H , et al. Role of adaptor TRIF in the MyD88‐independent toll‐like receptor signaling pathway. Science. 2003;301:640–3.1285581710.1126/science.1087262

3. Feng Y , Zou L , Zhang M , Li Y , Chen C , Chao W . MyD88 and Trif signaling play distinct roles in cardiac dysfunction and mortality during endotoxin shock and polymicrobial sepsis. Anesthesiology. 2011;115:555–67.2179205310.1097/ALN.0b013e31822a22f7PMC3162066

